# Anti‐PD‐1 therapy redirects macrophages from an M2 to an M1 phenotype inducing regression of OS lung metastases

**DOI:** 10.1002/cam4.1518

**Published:** 2018-05-07

**Authors:** Pooja Dhupkar, Nancy Gordon, John Stewart, Eugenie S. Kleinerman

**Affiliations:** ^1^ Division of Pediatrics The University of Texas M. D. Anderson Cancer Center Houston Texas; ^2^ Division of Pathology/Lab Medicine The University of Texas M. D. Anderson Cancer Center Houston Texas

**Keywords:** Immunotherapy, lung metastasis, macrophages, osteosarcoma, programmed death ligand‐1

## Abstract

Osteosarcoma (OS) pulmonary metastasis translates into poor patient survival. The implication of PD‐1‐PD‐L1 pathway in the context of NK cells and/or macrophages in OS is unknown. We investigated the effect of anti‐PD‐1 in OS lung metastasis and the role of NK cells and/or macrophages in anti‐PD‐1 responses. A human LM7 OS mouse model was used. Immunohistochemistry for tissues (PD‐L1, caspase‐3, Ki‐67, NK cells, macrophages), and Western blotting for OS lung tumors (p‐Stat3, p‐Erk1/2) was performed. NK and macrophages were assessed using flow cytometry. NK cell and macrophage depletion were conducted using anti‐asialo GM1 and clodrosome, respectively. PD‐L1 expression was observed in human OS cells and OS patient lung metastases. Anti‐PD1 antibody led to a significant decrease in the number of OS lung metastases, enhanced tumor apoptosis, decreased tumor cell proliferation, and p‐STAT‐3/p‐Erk1/2 signaling blockade in OS lung tumors. NK cells and macrophages in OS lung tumors expressed PD‐1 and anti‐PD1 increased NK cell and macrophage tumor infiltration. Increased numbers of antitumor M1 macrophages and decreased pro‐inflammatory M2 macrophages were seen. NK depletion did not affect therapeutic effect of anti‐PD‐1, suggesting that NK cells were not directly involved. However, macrophage depletion significantly compromised anti‐PD1 efficacy, confirming their role in efficacy of anti‐PD‐1 against OS lung metastasis. Our findings suggest that OS lung metastases regression by anti‐PD1 can be attributed to activated tumor M1 macrophages and reduced M2 macrophages. Owing to the co‐relation of M1 macrophages with OS patient outcome, we provide a novel mechanism of PD‐1 blockade and a basis for future clinical trials for anti‐PD‐1 antibodies in OS.

## Introduction

Osteosarcoma (OS) pulmonary metastasis is the main cause of patient mortality in OS, with the 5‐year patient survival rate being 25–30% 1. About 15–20% of the patients present with detectable lung metastases at diagnosis and have worse outcomes, urging the discovery of novel therapeutics 1. Immunotherapy has been promising to eradicate minimal residual and relapsed disease 2, 3, 4. Previously, we discovered that liposomal muramyl tripeptide phosphatidylethanolamine (L‐MTP‐PE), a macrophage/monocyte activator, showed efficacy in Phase II trial of relapsed OS lung metastasis patients and resulted in 8% improvement in the patient overall survival in Phase III trials 5, 6.

Programmed death receptor 1 (PD‐1), an immune‐inhibitory receptor on T, B, and NK cells, interacts with programmed death ligand 1 (PD‐L1), causing immunosuppression through decreased immune cell proliferation, cell survival, and cytolytic function 7. PD‐L1 upregulation has been known in melanomas, pancreatic, and other cancers 8, 9. The role of PD‐1 pathway in T cell‐mediated inhibition of antitumor responses has been studied. However, implication of PD‐1‐PD‐L1 pathway in the immunotherapeutic efficacy of NK cells and/or macrophages in OS has not been described. We hypothesize that checkpoint inhibitors offer benefit by blocking the immunosuppressive OS lung tumor microenvironment.

PD‐1 expression on mouse and human tumor‐associated macrophages (TAMs) has been shown to inhibit tumor immunity 10. Current studies are focusing on targeting TAMS in the tumor microenvironment, primarily suppressing the polarization from M1 macrophages to M2 phenotype 11, 12. Here, we investigated the effect of anti‐PD‐1 in OS lung metastases using a human LM7 OS mouse model, enabling the assessment of PD‐1 blocking effects on NK cells and macrophages, in the absence of T cells 13. We show that anti‐PD1 treatment induced NK and macrophage infiltration into OS lung metastases. In addition, increased migration of antitumor M1 macrophages and decreased migration of pro‐inflammatory M2 macrophages were observed. However, the therapeutic efficacy of anti‐PD1 was mediated by macrophages and not NK cells. Hence, we report that macrophages are crucial in the antimetastatic responses of anti‐PD1 in OS lung metastasis.

## Methods

### Cell lines

Human OS cell lines KRIB, U2OS, 143B, MG63.2, and SAOS‐2 cells were purchased from American Type Culture Collection (Rockville, MD). LM‐7 was derived from SAOS‐2 in our laboratory, and C‐CH‐OS‐D and C‐CH‐OS‐O (from primary patient tumor pretreatment biopsies) were provided by Dr. Hughes (M. D. Anderson) 13, 14. All cells were cultured in complete Dulbecco's modified Eagle's medium (Whittaker Bioproducts Inc., Walkersville, MD) and tested for mycoplasma using MycoAlert^™^ Mycoplasma Kit (Lonza, Allendale, NJ). For IFN‐γ treatment; OS cells were treated with IFN‐γ (200 U/mL) for 24 h.

### OS patient samples

Paraffin‐embedded tissues of lung metastases from 10 OS patients were obtained. The patient protocol was approved by IRB at M. D. Anderson Cancer Center.

### Animal model

Athymic female *nu/nu* mice purchased from National Cancer Institute (Bethesda, MD) were housed in an animal facility approved by American Association of Laboratory Animal Care. Protocols were in compliance with the Institutional Animal Care and Use Committee (IACUC) at M. D. Anderson Cancer Center. LM7 cells (2 × 10^6^) were intravenously injected in mice tail vein and anti‐mPD‐1 (RMPI‐14) (Bio X Cell [West Lebanon, NH]); treatment was started after 6–7 weeks, after confirmation of micrometastases formation by H&E staining of the lung tissues. Mice were randomly divided into two groups (five mice per group) and treated intraperitoneally (i.p.) with either PBS or anti‐PD‐1 antibody (200 μg/mouse) twice a week, for 5 weeks. Mice were sacrificed using 5% CO_2_, and major organs were resected. Tissues were either fixed with 10% formalin acetate (Fischer Scientific, Pittsburgh, PA) and paraffin‐embedded or were snap‐frozen in 1:1 Tissue‐Tek OCT (VWR, PA) and PBS.

### Toxicity studies

Analysis of mouse tissues was performed by a pathologist blinded to the study. Complete blood count (CBC) and chemistry profile were determined in serum for evidence of toxicity.

### Immunohistochemistry

Antigen retrieval was performed for tissues using 0.1 mol/L sodium citrate (pH 6.0) buffer or manufacturer's buffer. Sections were blocked using 3% H_2_O_2_, 4% fish skin solution (Electron Microscopy Sciences, Hatfield, PA) or goat‐serum (Sigma‐Aldrich, St. Louis, MO). Primary antibodies against h‐PD‐L1 (1:100) (Cell Signaling Technology, Danvers, MA), h‐Ki‐67 (1:100) (Neomarkers, Fremont, CA), cleaved caspase 3 (1:50) (Biocare Medical, Concord, CA), m‐F4/80 (1:200) and CD163 (1:100) (Abcam, Cambridge, MA), m‐NKp46 (1:50) (Biolegend, San Diego, CA), or CD68 (1:50) (BD Biosciences, San Jose, CA) were added overnight at 4°C. Anti‐rabbit‐IgG‐HRP (Santa Cruz Biotechnology, Dallas, TX) or anti‐rat‐IgG‐HRP (Jackson Immunoresearch, Westgrove, PA) secondary antibodies (1:1000) were used. 3, 3′‐diaminobenzidine (DAB) and hematoxylin counterstaining was performed followed by image capture (Leica Microsystems Inc., San Jose, CA) and quantification Simple PCI (Hamamatsu) 14.

### TUNEL staining

Paraffin‐embedded tissues were deparaffinized, proteinase K treated, and blocked with 3% H_2_O_2_ (Promega Corporation, Madison, WI). Sections were incubated with terminal transferase and biotin‐16‐dUTP (Roche Applied Sciences, Indianapolis, IN) for 1 h at 37°C, blocked with 2% bovine serum albumin, 5% normal horse serum, and Streptavidin‐HRP (Biocare, CA) for 30 min. DAB counterstain and hematoxylin counterstain were performed, and images were quantified using Simple PCI.

### Flow cytometry staining

Single cell lung tumors and spleen suspensions were obtained using 100‐μm syringe filter (Corning Inc., Corning, New York), treated with ACK buffer (Fisher Scientific, PA). Staining was performed with anti‐m‐F4/80‐APC and CD11b‐FITC (E‐Bioscience, San Diego, CA), anti‐mPD1‐PE and anti‐m‐NKp46‐PerCP (Biolegend, CA) antibodies, or isotype‐matched IgG controls for 30 min. FACSCalibur (Becton Dickinson, Mountain View, CA) was used (10,000 events per sample), and data were quantified by FlowJo (Ashland, OR).

### In vivo NK and macrophage depletion

For endogenous NK depletion, 50 μL anti‐asialo‐GM1 (Wako, VA) was i.p. injected into mice, twice weekly, and anti‐PD‐1 treatment was given after 24 h. Combination of anti‐asialo‐GM1 and anti‐PD‐1 treatment was performed for 5 weeks. Similar scheme was used for macrophage depletion studies. Liposomal clodronate (Encapsula NanoSciences, Brentwood, TN) was injected (200 μL, i.p., twice a week) followed by anti‐PD1 treatment 24 h later.

### Western blotting

Lung nodules were homogenized, lysed using RIPA (Santa Cruz, TX), and 10% SDS/PAGE was run after protein quantification (Bio‐Rad, Hercules, CA). Primary antibodies (1:1000): PD‐L1, p‐Stat3‐705, Stat‐3, phospho‐p44/42 MAPK (Thr202/Tyr204), p44/42 MAPK, cleaved caspase‐3 (Asp175), and caspase‐3 (Cell Signaling, MA) were used. Autoradiography detection and quantification were performed using Image J.

## Results

### PD‐L1 expression in OS cell lines

**Figure 1:**
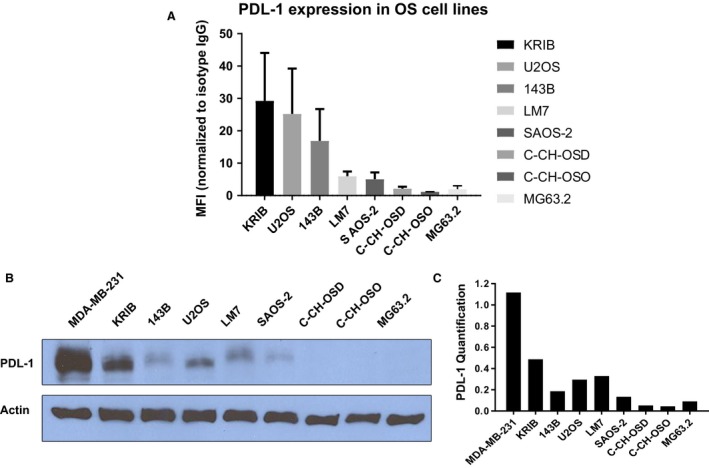
PDL‐1 is expressed in OS cell lines. Flow cytometry was performed using IgG‐APC or PDL‐1‐APC antibody. MFI (PDL‐1 positivity) normalized to IgG controls, standard deviations from three independent experiments are shown (A); Western blotting was performed using 10% SDS‐PAGE and anti‐hPDL‐1 antibody; MDA‐MB‐231 cells were positive control (B); ImageJ analysis was used for PDL‐1 (relative to actin) quantification (C).

Flow cytometry showed constitutive, variable surface PD‐L1 levels in human OS cells. PD‐L1 expression was highest in KRIB, U2OS, and 143 B‐cell lines (MFI: 29.2, 25.2, 16.8), intermediate‐high in LM7 and SAOS‐2 cell lines (MFI: 5.9 and 5.0) and lowest in C‐CH‐OS‐D, C‐CH‐OS‐O, and MG63.2 (MFI: 2.1, 1.1 and 1.9) (Fig. 1A). The total levels, evaluated by Western blotting, correlated with the surface levels (Fig. 1B and C). Visible total PD‐L1 protein levels were seen in KRIB, U2OS, 143 B, LM‐7, and SAOS‐2 using Western blotting, whereas very low levels were present in C‐CH‐OS‐D, C‐CH‐OS‐O, and MG63.2 cells as compared to MDA‐MB‐231 cells as the positive control.

We further demonstrated a significant increase in PD‐L1 expression in SAOS‐2, LM7, C‐CH‐OS‐D, and C‐CH‐OS‐O cells on IFN‐γ cytokine exposure (Fig. S1). Thus, PD‐L1 expression can be modulated by IFN‐γ in the in vivo tumor microenvironment.

### PD‐L1 expression in OS patient lung metastases

**Figure 2:**
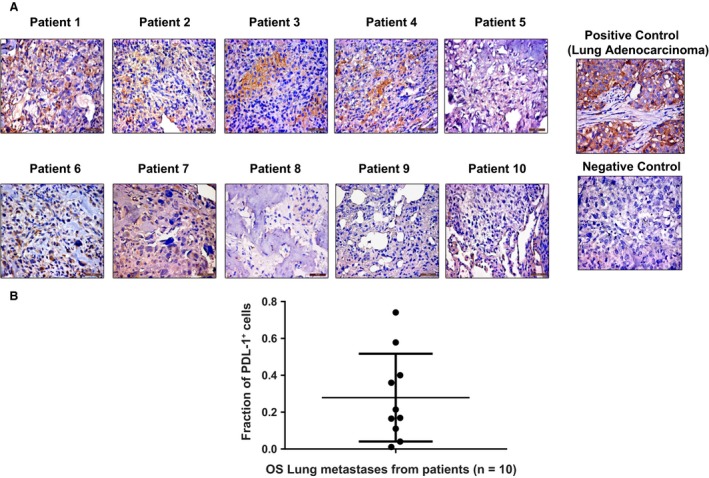
PDL‐1 is expressed in OS patient lung metastases. IHC staining was performed on paraffin‐embedded tissues for lung metastases from 10 patients with OS, using anti‐hPDL‐1 antibody and lung adenocarcinoma tissue as a positive control (A); quantification of PDL‐1 was analyzed by Cell Quest software and represented as fraction of PDL‐1 +  cells. Mean of PDL‐1 staining intensity of all patient samples is ~30% (B).

IHC staining of 10 OS patient lung metastasis paraffin‐embedded sections was performed using lung adenocarcinoma tissue as a positive control. We found PD‐L1 (PD‐L1^+^) expression in eight of 10 patients (membrane and cytoplasm) (Fig. 2A). Negative control showed no staining as well as positive control tissue showed high PD‐L1 staining intensity. Any staining (either cytoplasmic or membrane or both) was considered as PD‐L1 positive staining. Variable expression patterns were observed within the OS patient samples. Quantification confirmed an average of ~30% positive PD‐L1‐positive staining intensity (calculated based on the fraction of cells staining positive for PDL‐1 in every sample) (Fig. 2B). Our data demonstrated that PD‐L1 expression in OS lung metastases varies between patients.

### Effect of anti‐PD‐1 on OS pulmonary metastases, tumor cell proliferation, and apoptosis

**Figure 3:**
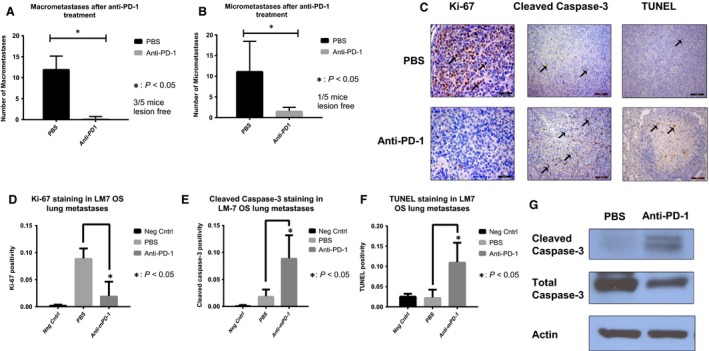
In vivo anti*‐*PD‐1 decreased LM7 lung metastases, tumor cell proliferation, and increased tumor apoptosis. Anti‐PD‐1 treatment (i.p., 200 μg) for 5 weeks significantly decreased the number of LM‐7 macrometastases (A) and micrometastases (B) (*P* < 0.05); representative IHC‐stained LM‐7 lung metastases sections using anti‐human‐Ki‐67 (Mag: 40×), cleaved caspase‐3 (Mag: 20×), and TUNEL (Mag: 40×) antibodies are shown (C); quantification for Ki‐67 (D), cleaved caspase‐3 (E), and TUNEL (F) positivity for three random microscopic fields using Cell Quest and Student's *t*‐test was performed (*P < 0.05). Decreased Ki67 and increased cleaved caspase‐3 and TUNEL staining indicate a decrease in tumor cell proliferation and an increase in apoptosis. (G) Western blotting from LM7 lung tumor nodules after anti‐PD‐1 5 week treatment showed increased cleaved caspase‐3 expression.

Anti‐mPD1 antibody was used to block the endogenous NK and macrophage PD‐1 and PD‐L1 interactions using our human LM‐7 OS mouse model 13. Due to 77% homology between murine and human PD‐L1, the binding affinities of murine PD‐1 (mPD‐1) to murine PD‐L1 (mPD‐L1) and mPD‐1 to human PD‐L1 (h‐PD‐L1) are equivalent 15, 16. Anti‐PD‐1 (200 μg/mouse) caused a significant decrease in the mean number of LM‐7 OS macrometastases and micrometastases (*P* < 0.05) after 5 weeks (Fig. 3A and B), demonstrating its efficacy against LM7 OS lung metastases.

To determine the anti‐PD1 response mechanisms, we assessed tumor cell proliferation (Ki‐67 IHC) and apoptosis (TUNEL). The number of Ki‐67^+^ tumor cells was significantly decreased in the anti‐PD‐1 group as compared to the control PBS group (*P* < 0.05) (Fig. 3C and D). In addition, cleaved caspase‐3 (Fig. 3C and E) and TUNEL expression (Fig. 3C and F) in anti‐PD‐1 group were significantly higher as compared to PBS controls. Further, Western blotting confirmed apoptosis induction through significant increase in cleaved caspase‐3 in lung tumors of anti‐PD‐1 group compared with the control (Fig. 3G). In conclusion, anti‐PD‐1 therapy decreased macro‐ and micrometastases by inhibiting tumor cell proliferation and increasing tumor cell apoptosis.

In addition, anti‐PD‐1 had a molecular effect on the OS lung tumor, through significant decrease in PD‐L1 expression, and its key regulators, p‐Stat3‐705, Stat3, and p‐Erk1/2 (Fig. S2). Thus, in vivo anti‐PD‐1 treatment inhibited the p‐Stat3/p‐Erk1/2 and PD‐L1 signaling in the LM7 tumors.

Pathological analysis of H‐ and E‐stained organ sections showed no toxicity or inflammation (data not shown). Anti‐PD1 did not cause significant changes in the complete blood cell counts and liver function in mice, indicating no significant systemic toxicities (Fig. S3). The normal range of WBC differential in nude mice is 4–7.4 × 10^3^/μL, which is in a comparable range of 4–11 × 10^3^/μL in humans, although the lymphocyte population is significantly less in nude mice and is composed of B cells in the majority with very few T cells 17.

### PD‐1 upregulation and increased NK cells and macrophages in OS lung tumors after anti‐PD‐1

**Figure 4:**
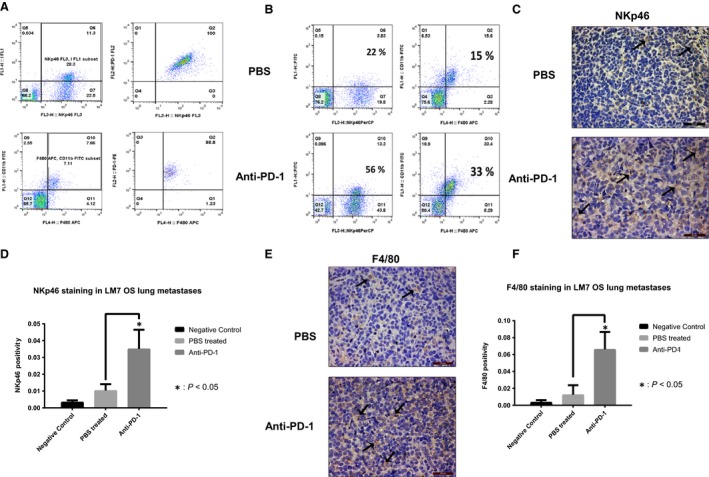
PD‐1 OS lung tumor expression and effect of anti‐PD‐1 on NK cells and macrophage infiltration. PD‐1 expressing NK cells and macrophages were identified by flow double staining using anti‐PD‐1‐PE with anti‐NKp46‐PerCP or anti‐F4/80‐APC and anti‐CD11b‐FITC antibodies (A); LM‐7 tumor cell suspensions were analyzed for infiltrating NK cell and macrophages by flow NKp46 and F4/80, CD11b staining (B); NK cells were assessed in LM7 metastasis sections with IHC NKp46 staining (C) and NKp46 quantification. * indicates *p* < 0.05.

PD‐1 expression on NK cells and macrophages in OS tumors is unknown. Flow cytometry of OS lung tumor cell suspension demonstrated that 98.8% of macrophages (F4/80^+^/CD11b^+^) and 100% of the NK (NKp46^+^) cells were PD‐1 positive (Fig. 4A). These findings support a potential immune escape mechanism for OS tumor cells.

To analyze anti‐PD1 activated immune responses, we quantified NK and macrophage infiltration within the OS lung tumors. Flow cytometry revealed a 2.5‐fold increase in the number of NK cells (NKp46^+^: 22–56%) and a 2.6‐fold increase in the number of macrophages (F4/80^+^/CD11b^+^: 15–33%) in the anti‐PD‐1 group compared with the control, respectively (Fig. 4B). However, NK cells and macrophages in spleens were unchanged after anti‐PD‐1 (Fig. S4). This data reflected that in the absence of T cells, the effect of anti‐PD‐1 on NK and macrophage infiltration was specific to the OS lung tumor site.

NKp46 and F4/80 IHC staining were significantly increased in LM7 OS lung metastases of anti‐PD‐1 group than the controls, primarily inside the tumor compared with the tumor periphery (Fig. 4C–F). These results support our flow data of increased number of NK cells and macrophages infiltrating into the OS lung tumors after anti‐PD1 treatment in mice in the absence of T‐cell population.

### Anti‐PD1 increases M1 and decreases M2 macrophages in OS lung tumors

**Figure 5:**
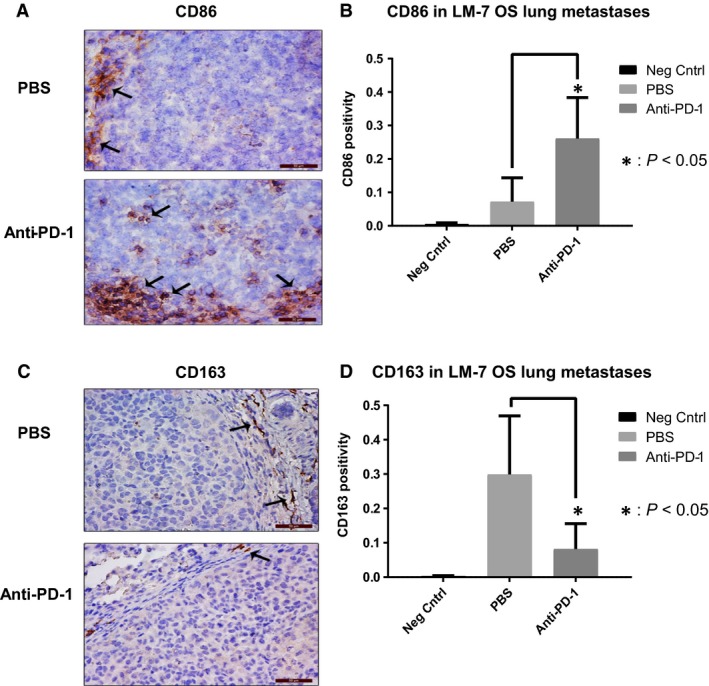
Effect of anti‐PD‐1 on M1 and M2 macrophages in LM7 lung metastases. CD86 IHC staining was performed on lung metastases sections (A); quantification with Cell Quest software and Student's *t*‐test was performed *P < 0.05 (B); CD163 IHC staining was performed on lung metastases tissues (C); quantification with Cell Quest software and Student's *t*‐test was performed, (Mag: 40×), *P < 0.05 (D).

CD86 IHC staining for M1 macrophages revealed a significant increase in the M1 macrophages within LM7 OS lung metastases after anti‐PD1 treatment (*P* < 0.05) (Fig. 5A and B). By contrast, CD163^+^ M2 macrophages were observed in the tumor periphery and were significantly decreased in the anti‐PD1‐treated mice compared with controls (*P* < 0.05) (Fig. 5C and D). Thus, PD‐1 blockade shifts the balance of pro‐inflammatory M2 macrophages toward activated antitumorigenic M1 macrophages, which may play a role in its’ therapeutic efficacy.

### The antitumor efficacy of anti‐PD1 is mediated by macrophages and not NK cells

**Figure 6:**
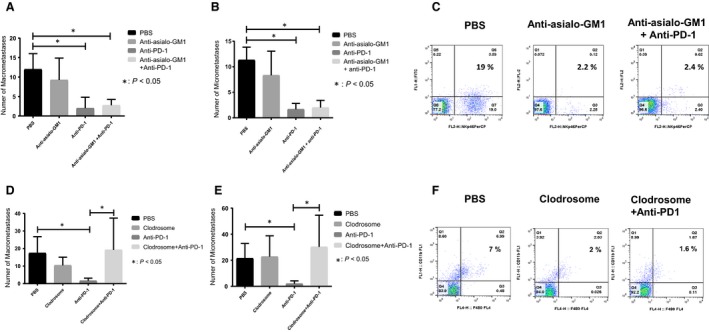
Effect of NK cell or macrophage depletion on anti‐PD‐1 efficacy against LM7 lung metastases. The number of OS lung macrometastases (A) and micrometastases (B) was counted after 5 week treatment with PBS, anti‐asialo‐GM1 (50 μL, twice weekly), anti‐PD‐1 (i.p. 200 μg/mouse), or both; NK depletion efficacy was determined with flow cytometry for LM‐7 lung tumor suspensions using anti‐NKp46‐PerCP or isotype IgG control antibody (C); the number of OS lung macrometastases (D) and micrometastases (E) was determined after 5‐week treatment of PBS, clodrosome (200 μL/mouse), anti‐PD‐1 (i.p., 200 μg/mouse), or combination; macrophage depletion was assessed by flow cytometry of lung tumor cell suspensions from treatment groups using anti‐CD11b‐FITC and anti‐F4/80‐APC antibodies. Student's *t*‐test was used for analysis (*P < 0.05) (F).

To investigate if NK cells are crucial in anti‐PD‐1 response, NK cell depletion was performed using anti‐asialo GM1 and confirmed in lung tumors (Fig. 6C) and spleens (Fig. S5). NK depletion had no effect on the anti‐PD‐1 antimetastatic activity (Fig. 6A and B). Mice treated with anti‐PD1 or anti‐asialo GM1 preceding anti‐PD1 had a similar response compared with the controls (*P* < 0.05). Thus, the therapeutic effect of anti‐PD1 was not compromised on NK depletion. In addition, NK depletion did not affect the growth of lung metastases. Hence, NK cells were not critical in the anti‐PD‐1 efficacy against LM7 lung metastases.

To assess the role of macrophages in anti‐PD1 response, macrophage depletion was performed. Clodrosome treatment caused a significant decrease in macrophages (CD11b^+^F480+) in OS lung tumors and spleens (Figs. 6F and S6), but not NK cells. The number of macro‐ and micrometastases was significantly compromised following macrophage depletion (Fig. 6C and D). The number of metastases in mice pretreated with clodrosome followed by anti‐PD1 was similar to the PBS group (*P* > 0.05). Clodrosome did not affect the number of metastases. These data, along with findings from Fig. 6, suggest that macrophages are responsible for anti‐PD1 antitumor responses in LM7 OS model.

## Discussion

PD‐1 inhibitors have shown promise in the clinic 18. Most in vivo studies have discovered T‐cell activation as the primary mechanism of anti‐PD1 responses 9, 19. Here, we demonstrate that anti‐PD‐1 antibody mediated OS lung metastasis regression through macrophage activation, through increased M1 and decreased M2 macrophage infiltration.

Tumor PD‐L1 upregulation has been known to block antitumor immune responses 20. Our data of in vitro PD‐L1 expression and variability in OS cell lines are similar to previous reports in melanoma 21. We also show that PD‐L1 is expressed in OS patient lung metastases and primary tumors. Recently, PD‐L1 expression was demonstrated in 75% of OS metastatic patients, but not in primary OS patients 22. We observed PD‐L1 expression in both primary tumors and lung metastases. The variable PD‐L1 expression in patients may be due to the heterogeneity, as observed in non‐small‐cell lung cancer (35–95%) and melanoma (40–100%) 8, 23. Importantly, anti‐PD‐1 patient responses may not depend on strong PD‐L1 positivity as discovered in non‐small‐cell lung cancer patients 18. Thus, our data support that PD‐L1 is a potential OS therapeutic target in the clinical setting.

PD‐1/PD‐L1 inhibitors have shown efficacy in preclinical melanoma and pancreatic cancer models 9, 19, 24. We discovered that anti‐PD1 caused regression of OS lung metastasis. Due to the significant homology, the binding affinities of mPD‐1 to mPD‐L1 and mPD‐1 to h‐PD‐L1 are equivalent 15, 16. Hence, in our model, antimurine PD‐1 antibody likely exerts therapeutic efficacy due to blockade of mPD‐1 on endogenous mouse NK cells/macrophages binding with hPDL‐1 on LM7 tumor cells. Our data align with previous study showing reduced OS metastases after PD‐L1 blockade 22. In addition, we uncover efficacy mechanisms including inhibition of tumor cell proliferation, apoptosis induction, inhibition of p‐Stat3/PD‐L1 pathway. We also established the safety of anti‐PD‐1 at the administered dosage.

PD‐1 upregulation on tumor‐infiltrating T cells has been associated with T‐cell dysfunction 7. PD‐1 is induced on activated NK cells in infection, multiple myeloma and on macrophages in sepsis 25, 26. Also, that mouse and human TAMs have elevated PD‐1 expression and that high expression inhibits macrophage phagocytosis and tumor immunity 10. Here, we demonstrated for the first time that PD‐1 is upregulated on both NK cells and macrophages in OS lung metastases. This indicates suppressed NK and macrophage function, which may contribute to an immunosuppressive OS lung metastatic microenvironment. Although previous research has shown tumor T‐cell infiltration after PD‐1/PD‐L1 blockade, the importance of macrophages and *intrinsic* immune responses in tumor immunity and therapy efficacy is now being recognized 22. Recent evidence demonstrated that checkpoint inhibitors enhance NK cell‐mediated antimetastatic activity against lung metastasis and are not limited to only T cells 27. Our nude mouse model allowed us to focus on the specific effects of anti‐PD‐1 on NK cells and macrophages. Our data showing increased NK and macrophage tumor infiltration after anti‐PD‐1 treatment are unique and entail targeting PD‐1 on the innate immune system, as opposed to PDL‐1 on the tumors. The effect of anti‐PD‐1 in an immune‐competent mouse model needs to be further evaluated to understand T‐cell contribution.

Our discovery of increased M1 macrophages and decreased M2 macrophages on PD‐1 blockade aligns with previous literature of L‐MTP‐PE causing M1 activation, cytokine release, and OS cell death 5, 28. Tumor‐infiltrating macrophages have been associated with OS metastasis suppression. Recent studies have shown that M2 macrophages promoted breast cancer metastasis progression 29. M2 macrophage depletion using CSF‐1R inhibitor has been found to enhance dendritic cell immunotherapy responses 30. M1/M2 macrophage polarization (increase in M1 versus M2 macrophages) has been co‐related with increased survival of non‐small‐cell lung cancer patients 12. Thus, suppressing M1 to M2 macrophage polarization is being investigated as a potential therapeutic approach. In conclusion, our data showing an increase in M1 and decrease of M2 macrophages, possibly through macrophage polarization, uncover a unique mechanism of anti‐PD1.

Unlike other cancers, TAMs play an antimetastatic role in OS, as the infiltrating TAMs have been shown to correlate with better patient survival 31. Strikingly, we discovered that macrophages were predominant effector cells involved in anti‐PD‐1 antitumor responses. Recently, Gordon et al. 10 also revealed decreased colorectal tumor growth on PD‐1‐PD‐L1 blockade, in a macrophage‐dependent manner, through enhanced phagocytosis. Activated macrophages cause induction of iNOS (inducible NO synthase), NO (Nitric Oxide), or cytokine release [TNF‐α, IL‐6], causing decreased tumor growth and metastasis 28.

Despite increased NK cells migration in tumors, we demonstrated that NK cells were not crucial for anti‐PD‐1 efficacy. By contrast, the anti‐PD‐1 efficacy was lost in macrophage‐depleted mice. Recent studies of L‐MTP‐PE and IL‐2/IL‐15 showed that macrophages can be directly cause phagocytosis, independent of NK cells 28, 32. Also, PD‐1/PD‐L1 blockade in TAMs led to direct macrophage‐led phagocytosis of tumor cells 10. Our data indicate that NK cells were not critical in direct killing of OS tumor cells. It is possible, however, that NK cells may release cytokines causing M1 activation and the killing of M2 macrophages 33.

Our observations have a translational significance for relapsed OS patients, with lymphocyte immunosuppression postchemotherapy. Previously, low absolute lymphocytes were found in sarcoma patients after prolonged chemotherapy 34. However, the macrophage cytotoxic function is not suppressed by chemotherapy for newly diagnosed/relapsed disease OS patients 35. Hence, our results indicate that anti‐PD‐1 can be combined with chemotherapy in newly diagnosed patients to eradicate lung micrometastases, thus improving survival. Anti‐PD‐1 alone or in combination with chemotherapy may eradicate lung metastases in relapsed patients through macrophage activation, and by increasing the M1/M2 ratio.

In summary, targeting PD‐1/PD‐L1 signaling is a promising approach for the treatment of OS lung metastasis. Notably, macrophages are the key mediators of anti‐PD1 efficacy, and redirecting M2 macrophages to M1 resulted in regression of OS lung metastasis. These novel mechanisms provide a strong rationale for clinical translation of anti‐PD‐1 antibodies alone or in combination with other immunotherapies for the treatment of OS lung metastasis patients for improved clinical outcomes.

## Conflict of Interest

The authors declare no competing interests.

## Supporting information


**Figure S1**. IFN‐γ induces PDL‐1 expression in OS cells. IFN‐γ (200 U/mL) 24 h treatment was performed followed by flow cytometry using anti‐PDL‐1‐APC antibody or IgG‐APC isotype control. Mean, standard deviations for PDL‐1 positivity shown (*P < 0.05).

**Figure S2**. Anti‐PD‐1 inhibits p‐Stat3/PDL‐1 pathway in LM7 lung tumors. PDL‐1 IHC staining (A) and Quantification (B) was performed on LM7 sections using anti‐hPDL‐1 antibody, Mean ± SD of PDL‐1 positivity was calculated and student's *t*‐test was performed, *P < 0.05; Western blot (C) and Image J quantification (D) was performed for LM7 lung tumors after 5 week treatment, Actin was the loading control.

**Figure S3**. Anti‐PD‐1 did not change CBC and liver function. Hemoglobin, hematocrit, platelets and WBC counts were determined in mice blood after 5 week treatment (A); AST and ALT liver enzymes were evaluated from mice serum. Student's *t*‐test was used for analysis (*P* > 0.05) (B).

**Figure S4**. Anti‐PD‐1 treatment did not affect NK cells and macrophages in spleen. Flow cytometry was performed on lung tumor suspensions using anti‐NKp46‐PerCP or anti‐F4/80‐APC and anti‐CD11b‐FITC antibodies.

**Figure S5**. Anti‐asialo‐GM1 significantly decreased NK cells in spleen. Flow cytometry was performed for spleen cell suspensions after anti‐asialo‐GM1 (50 μL, twice weekly) treatment using anti‐NKp46‐PerCP or anti‐F4/80‐APC and anti‐ CD11b‐FITC antibodies.

**Figure S6**. Clodrosome significantly decreased spleen macrophages. Flow cytometry was performed with spleen cell suspensions after Clodrosome (200 μL, twice weekly) treatment using anti‐F4/80‐APC and anti‐CD11b‐FITC or anti‐NKp46‐PerCP antibodies.
