# Supplementary figures and images for: Anti‐PD‐1 therapy redirects macrophages from an M2 to an M1 phenotype inducing regression of OS lung metastases
Source: Cancer Med. 2018 May 7;7(6):2654–64. doi: 10.1002/cam4.1518 (PMC6010882; doi:10.1002/cam4.1518)

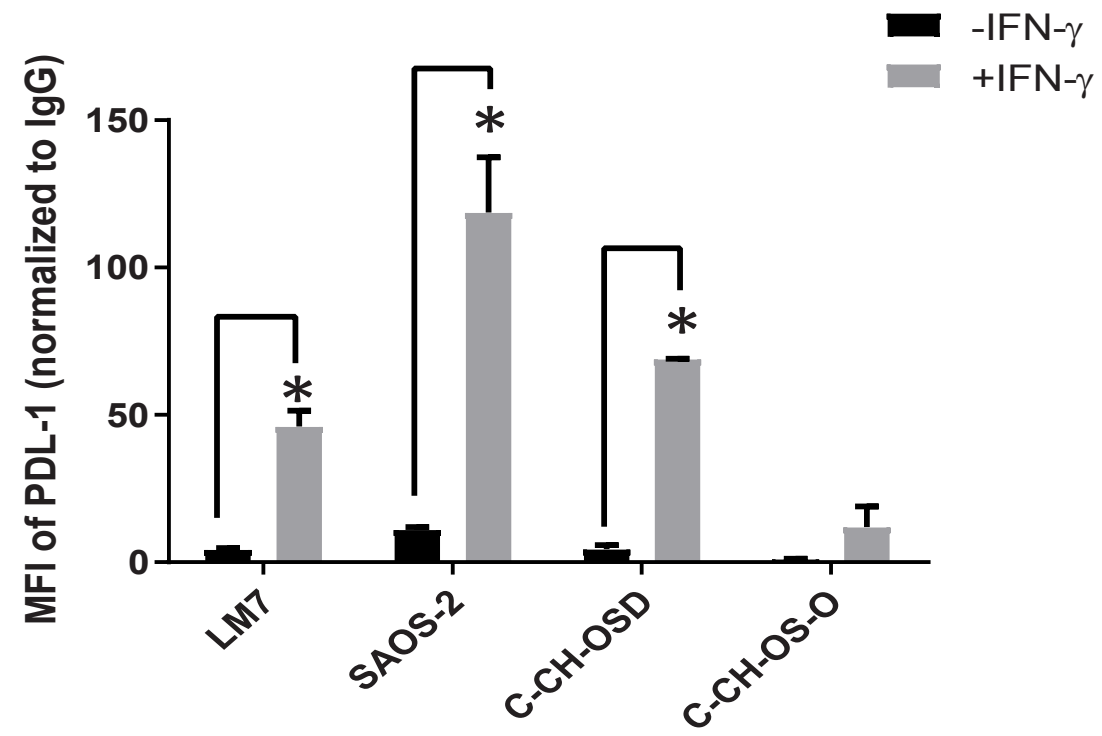

Supplement: Supplementary file 1 — Figure S1. IFN‐γ induces PDL‐1 expression in OS cells. IFN‐γ (200 U/mL) 24 h treatment was performed followed by flow cytometry using anti‐PDL‐1‐APC antibody or IgG‐APC isotype control. Mean, standard deviations for PDL‐1 positivity shown (*P < 0.05). [file CAM4-7-2654-s001.pdf]

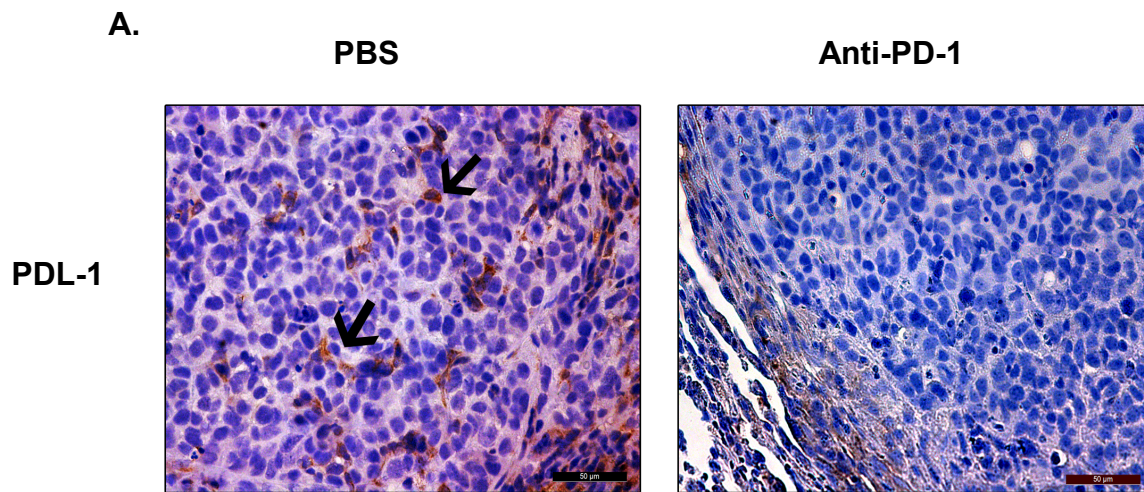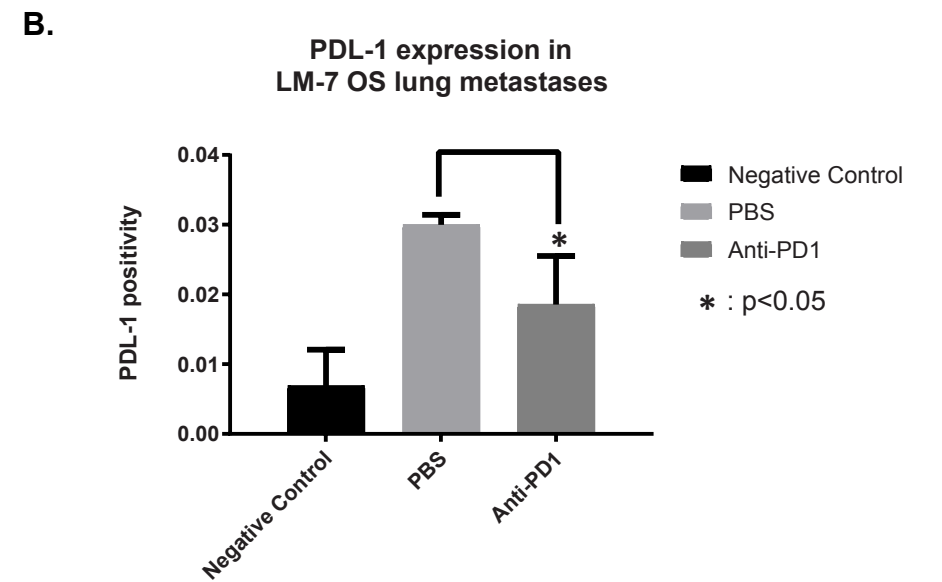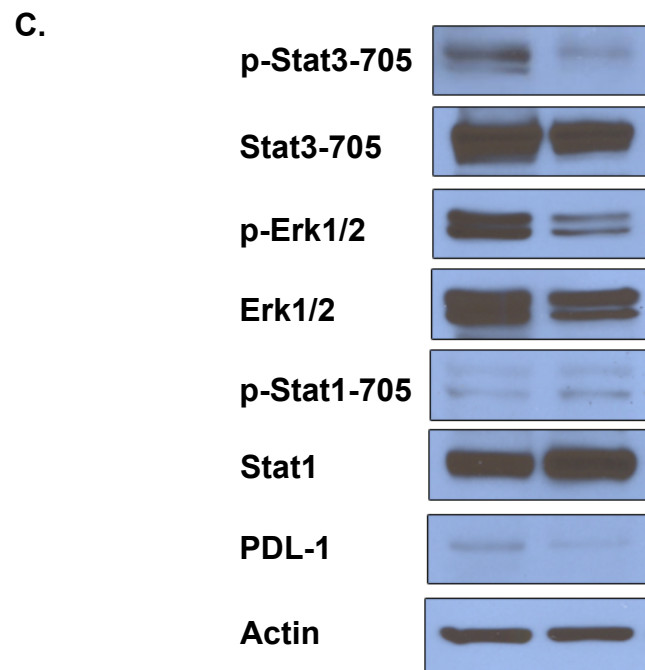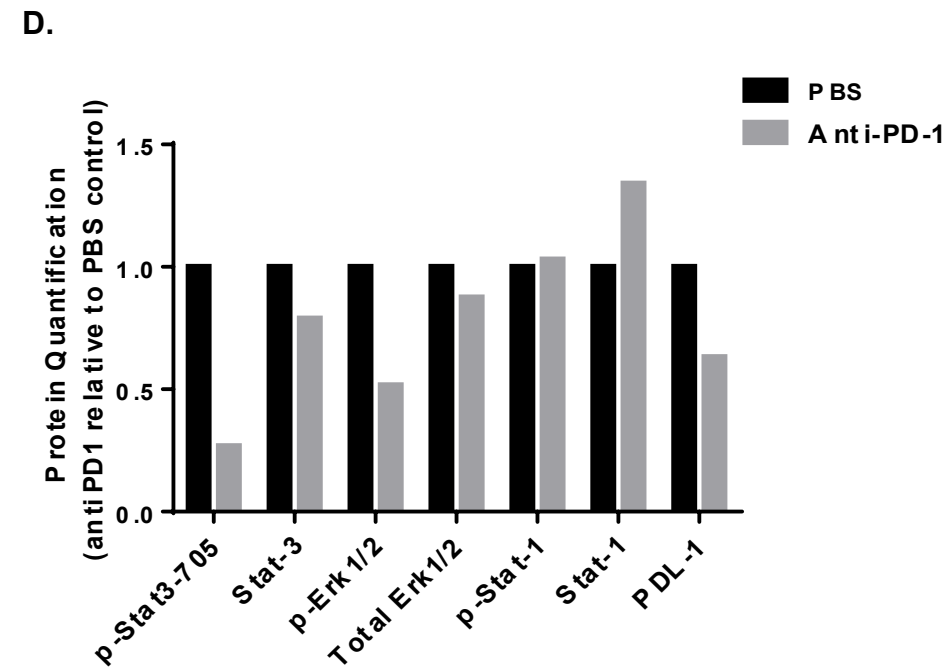

Supplement: Supplementary file 2 — Figure S2. Anti‐PD‐1 inhibits p‐Stat3/PDL‐1 pathway in LM7 lung tumors. PDL‐1 IHC staining (A) and Quantification (B) was performed on LM7 sections using anti‐hPDL‐1 antibody, Mean ± SD of PDL‐1 positivity was calculated and student's t‐test was performed, *P < 0.05; Western blot (C) and Image J quantification (D) was performed for LM7 lung tumors after 5 week treatment, Actin was the loading control. [file CAM4-7-2654-s002.pdf]

**A.**

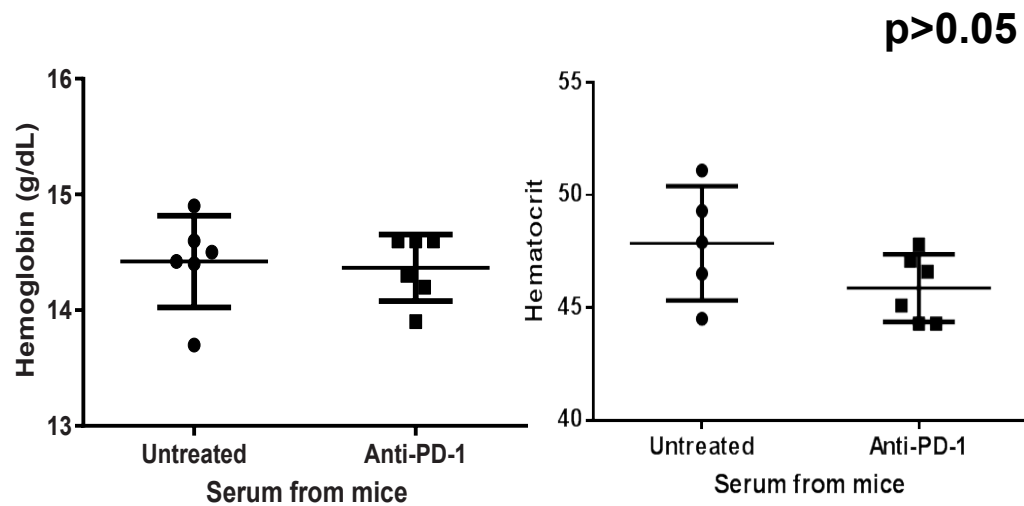

**B.**

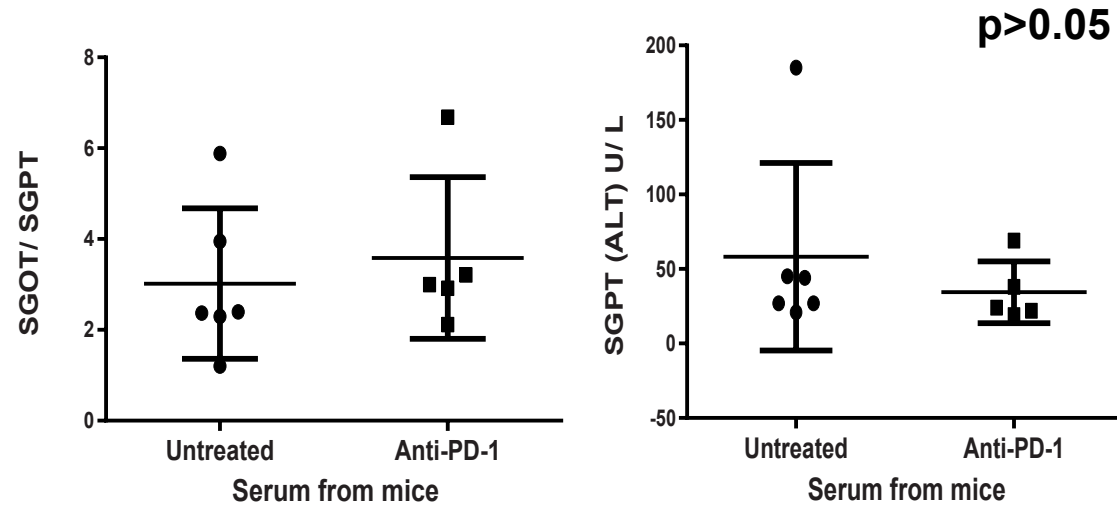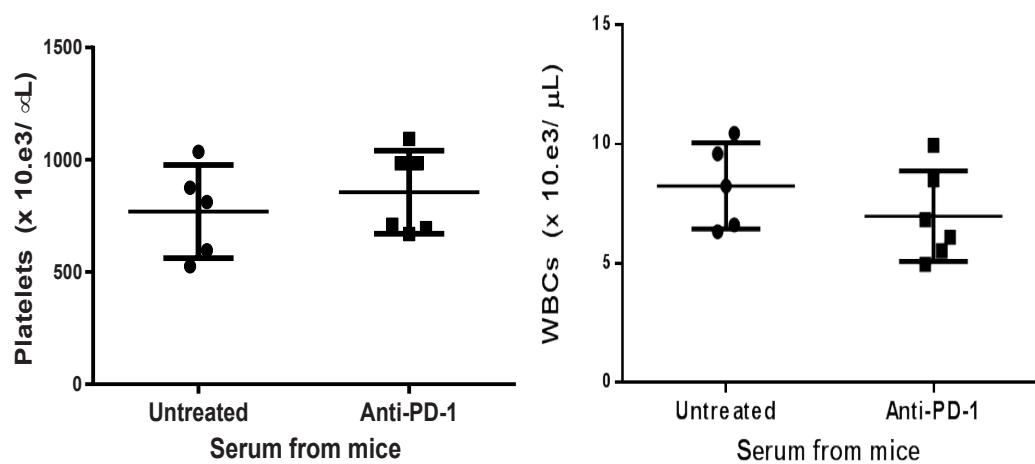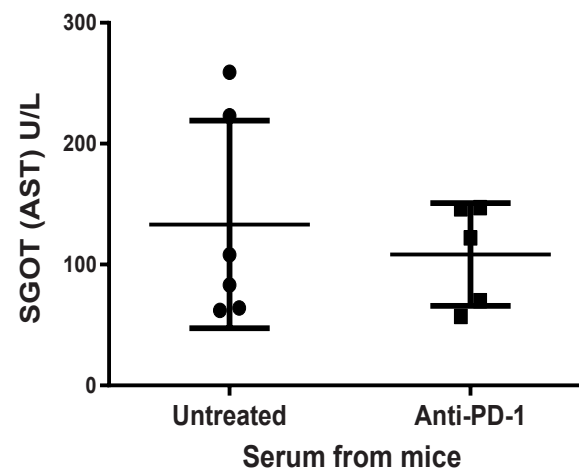

Supplement: Supplementary file 3 — Figure S3. Anti‐PD‐1 did not change CBC and liver function. Hemoglobin, hematocrit, platelets and WBC counts were determined in mice blood after 5 week treatment (A); AST and ALT liver enzymes were evaluated from mice serum. Student's t‐test was used for analysis (P > 0.05) (B). [file CAM4-7-2654-s003.pdf]

PBS

NKp46

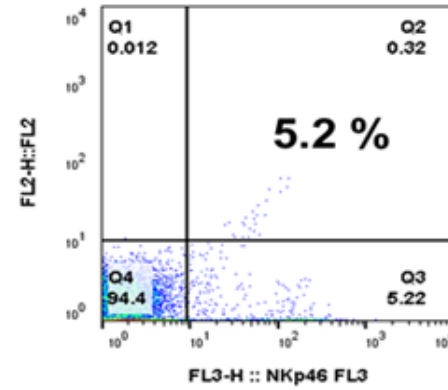

F4/80

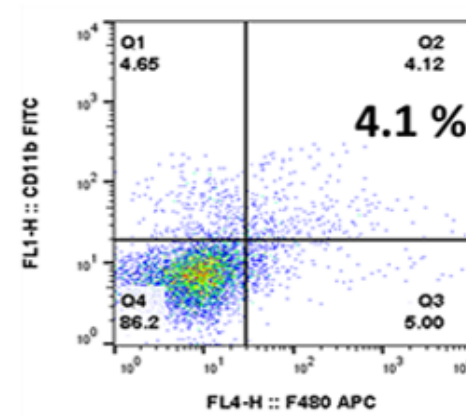

Anti-PD-1

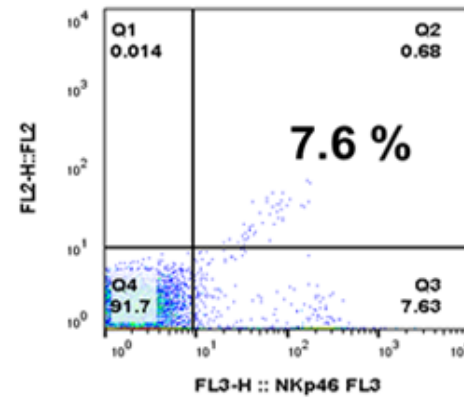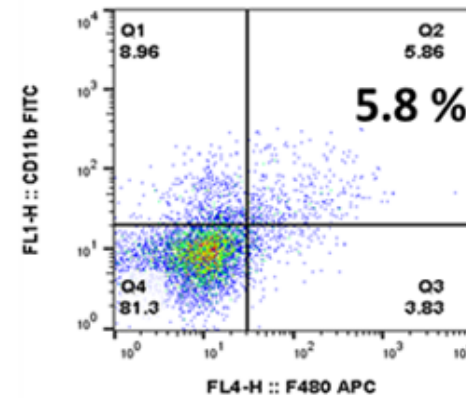

Supplement: Supplementary file 4 — Figure S4. Anti‐PD‐1 treatment did not affect NK cells and macrophages in spleen. Flow cytometry was performed on lung tumor suspensions using anti‐NKp46‐PerCP or anti‐F4/80‐APC and anti‐CD11b‐FITC antibodies. [file CAM4-7-2654-s004.pdf]

PBS

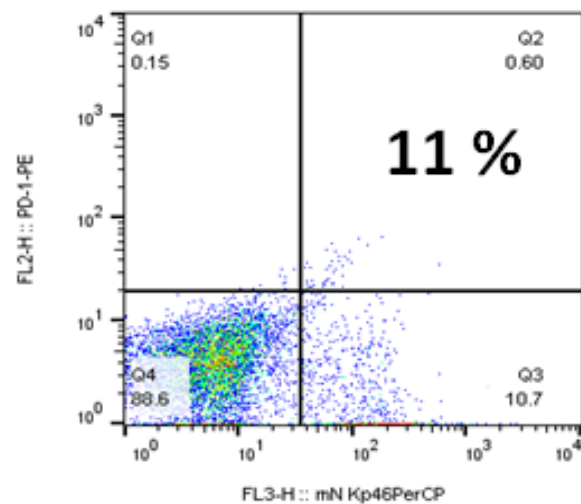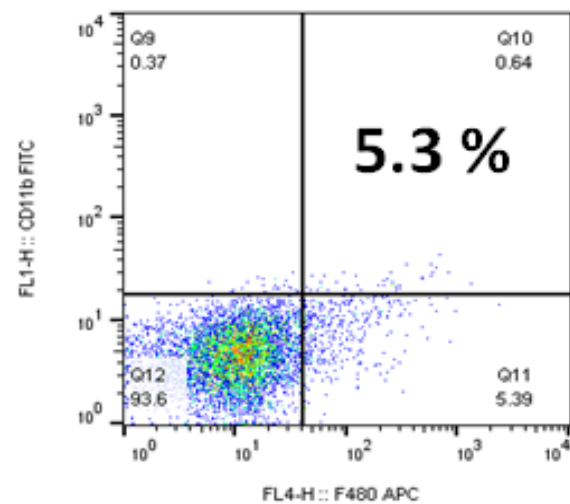

Anti-asialo-GM1

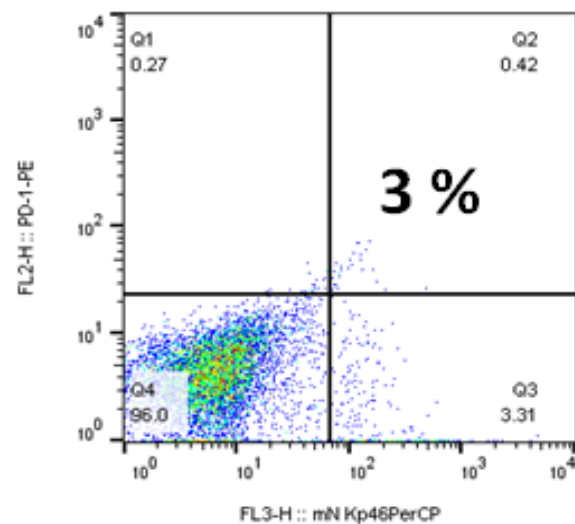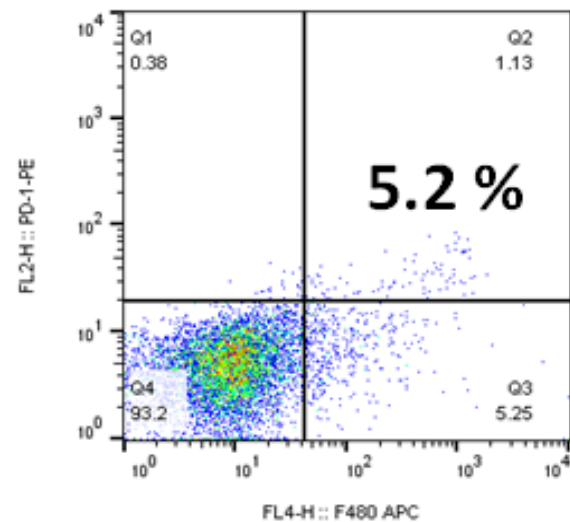

Supplement: Supplementary file 5 — Figure S5. Anti‐asialo‐GM1 significantly decreased NK cells in spleen. Flow cytometry was performed for spleen cell suspensions after anti‐asialo‐GM1 (50 μL, twice weekly) treatment using anti‐NKp46‐PerCP or anti‐F4/80‐APC and anti‐ CD11b‐FITC antibodies. [file CAM4-7-2654-s005.pdf]

PBS

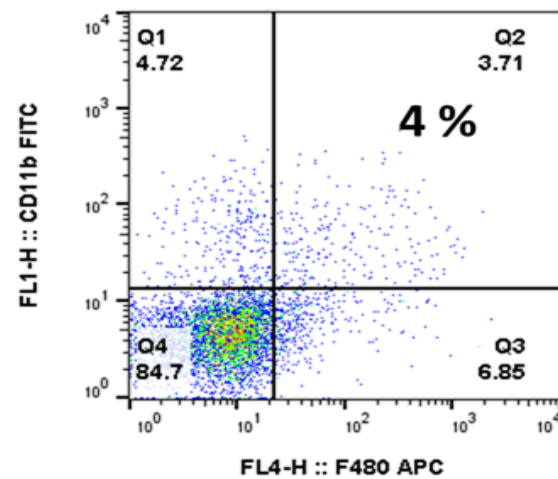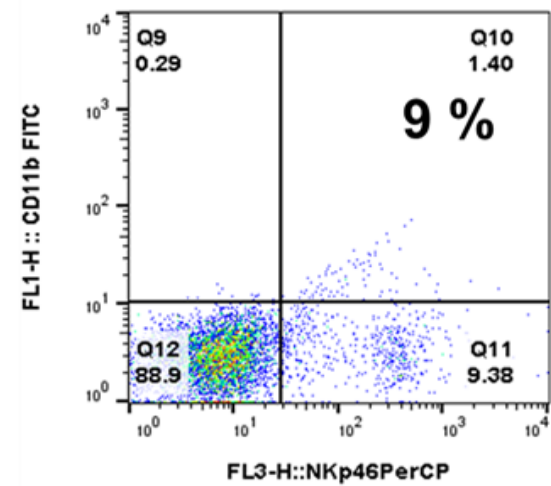

Clodrosome

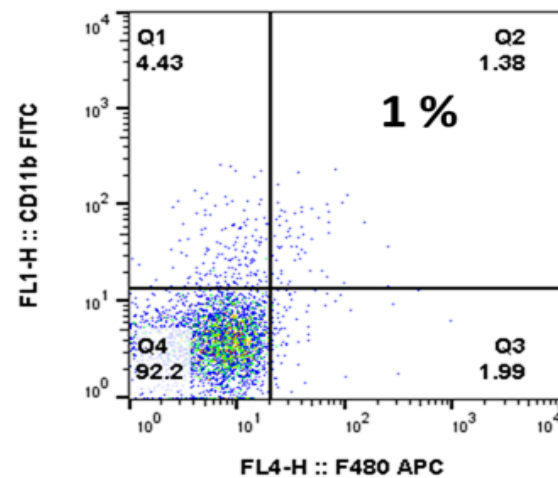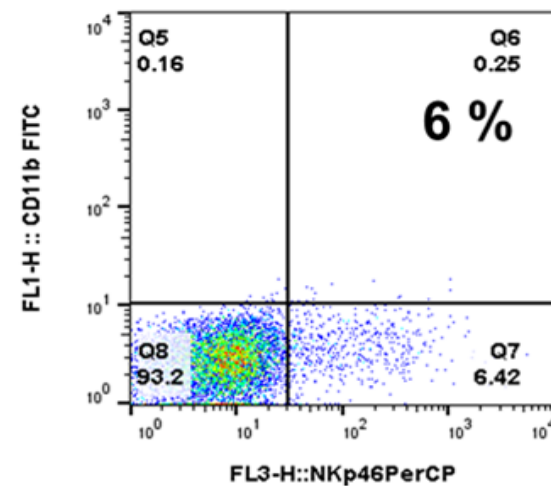

Supplement: Supplementary file 6 — Figure S6. Clodrosome significantly decreased spleen macrophages. Flow cytometry was performed with spleen cell suspensions after Clodrosome (200 μL, twice weekly) treatment using anti‐F4/80‐APC and anti‐CD11b‐FITC or anti‐NKp46‐PerCP antibodies. [file CAM4-7-2654-s006.pdf]
